# Combined Reconstruction of the Posterolateral Corner, Anterior Cruciate Ligament, and Lateral Extra‐articular Tenodesis Using Autografts and a Single Femoral Tunnel

**DOI:** 10.1002/atn2.70001

**Published:** 2026-05-24

**Authors:** Igor Natário Pinheiro, Rafael Erthal de Paula, Igor Stefano Menescal Pedrinha, João Gabriel de C. C. Villardi, Nathan Silvério Lopes Ramos, Rafael Moraes de Castro Padovani, Vitor Chatack de Oliveira, Gustavo de Matos Pinto Bravo, Kassio Emanuel Loureiro Cravo, Rossano Fiorelli, Alfredo Marques Villardi

**Affiliations:** ^1^ Orthopaedic Division Hospital Universitário Gaffrée e Guinle Rio de Janeiro Brazil; ^2^ Knee Specialized Attendance Center National Institute of Traumatology and Orthopedics (INTO) Rio de Janeiro Brazil

## Abstract

This surgical technique describe the reconstruction of the anterior cruciate ligament and the posterolateral corner, in addition to lateral extra‐articular tenodesis, using a single femoral tunnel positioned at Stannard's isometric point. The technique employs autologous grafts from the semitendinosus, gracilis, and iliotibial band, resulting in a more cost‐effective, faster, and reproducible procedure compared to isolated anterior cruciate ligament and posterolateral corner reconstruction.

## Graphical abstract

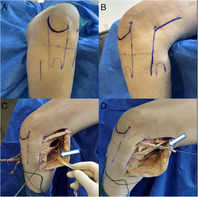

**VIDEO 1:** Technique for combined reconstruction of the anterior cruciate ligament and the Posterolateral Corner, associated with lateral extra‐articular tenodesis (LET), using a single femoral tunnel positioned at Stannard's isometric point. The technique employs autologous semitendinosus, gracilis, and iliotibial band grafts. Video content can be viewed at https://doi.org/10.1002/atn2.70001.

Multiligament knee injuries represent a significant clinical challenge, often resulting from traumatic dislocations. Data indicate that 12.1% of cases involve isolated injuries to the anterior cruciate ligament (ACL) and the posterolateral corner (PLC), while 30.4% involve combined injuries to the ACL, PLC, and posterior cruciate ligament (PCL). Additionally, 7.3% of injuries affect all ligaments ACL, PCL, PLC, and medial collateral ligament which leads to the need for ACL and PLC reconstruction in approximately 49.8% of cases.^1^

The anatomy of the PLC includes the fibular collateral ligament (FCL), the popliteus tendon, and the popliteofibular ligament, which are fundamental in restraining various angulation and external rotation.^2^, ^3^ The popliteus muscle acts as the primary restrictor of external tibial rotation and as a secondary restrictor of varus and posterior translation. The ACL is the primary limiter of anterior translation and acts as a secondary restrictor of varus and rotational movements.^3^, ^4^, ^5^, ^6^ Given the complexity of these combined injuries, it is necessary to simultaneously reconstruct the ACL and the PLC.^2^, ^3^, ^4^, ^5^, ^6^

Conventional techniques often utilize allografts and require two to three tunnels in the lateral femoral condyle, which increases the risk of tunnel collision, trochlear injury, fractures, and osteonecrosis.^7^, ^8^, ^9^, ^10^, ^11^ LaPrade et al. reported that the distance between the femoral insertions of the FCL and popliteus tendon is approximately 18.5 mm.^8^ Kim et al., conducted in a Korean population, show that this distance can be 9.3 mm.^12^

Angelini et al. described a technique using a single femoral tunnel for combined ACL and PLC reconstruction, employing autologous semitendinosus (ST) and gracilis (G) grafts; this technique requires grafts with a minimum length of 27 cm.^13^

Given these limitations, the aim is to describe a surgical technique for combined ACL and PLC reconstruction utilizing autologous grafts, which offers enhanced rotational stability in the lateral compartment, minimizes complications, and reduces overall procedural costs. This advantages and disadvantages of this technique are explained in Table 1.

**TABLE 1:** Advantages and Disadvantages of the Surgical Technique

**Advantages**	**Disadvantages**
Use of autologous grafts	Requires bilateral lower limb access for graft harvesting
Reduction in overall procedural costs	Increased morbidity due to autologous graft harvesting
Decreased risk of tunnel convergence	Interdependence between ACL and posterolateral corner reconstructions
Lower risk of trochlear cartilage injury during the preparation of femoral tunnels	Single tunnel at lateral condyle for the PLC and LET may not at the anatomical position
Reduced risk of lateral femoral condyle osteonecrosis	No clinical results data
Incorporates anterolateral rotational stability	Requires a more extensive lateral surgical approach

ACL, anterior cruciate ligament; LET lateral extra‐articular tenodesis; PLC, posterolateral corner.

## SURGICAL TECHNIQUE

The surgical technique described involves the combined reconstruction of the PLC, ACL, and LET, using autografts and a single femoral tunnel (Video 1).

**FIGURE 1:**
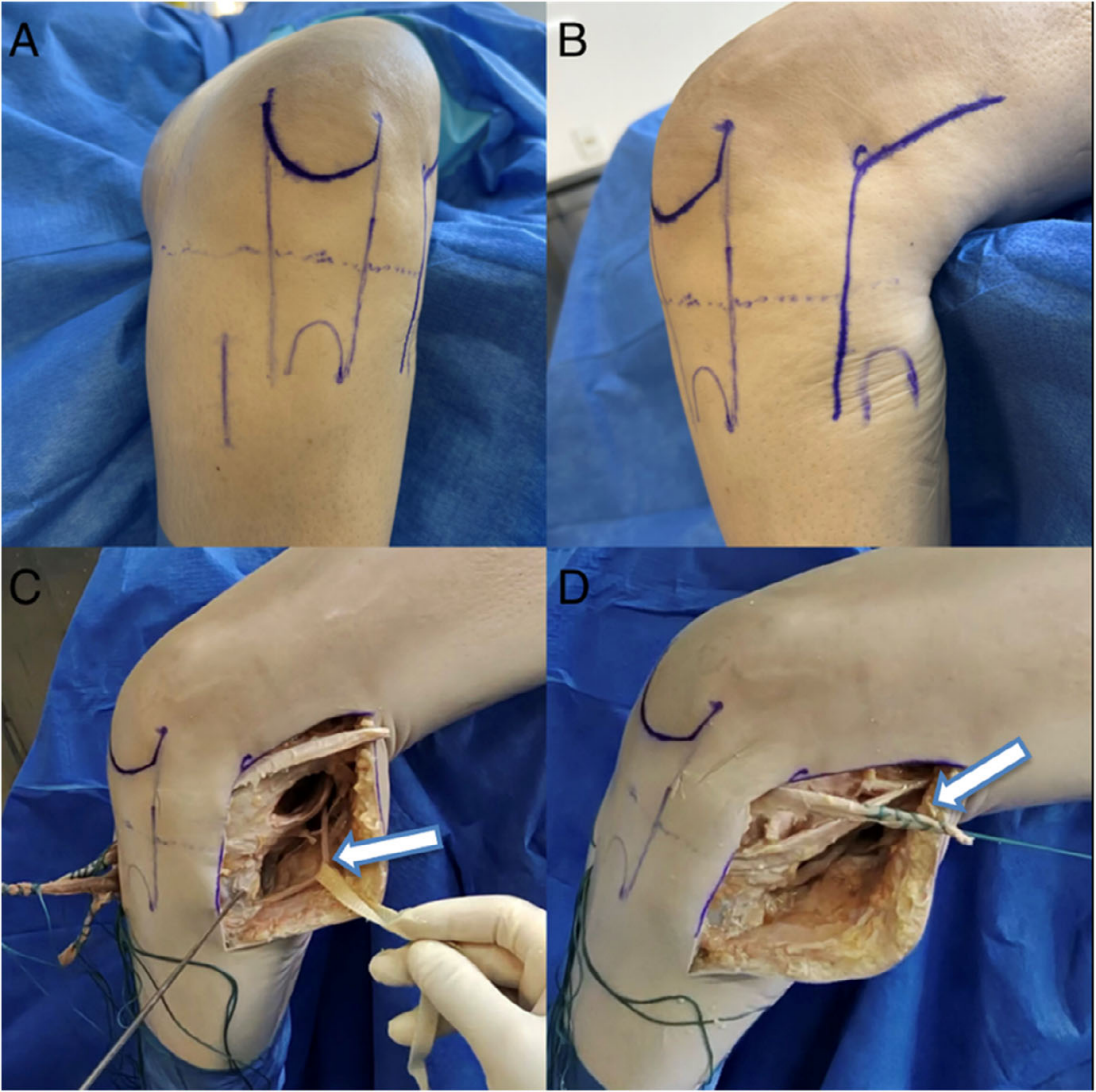
First steps of surgical procedure for treating multiligament knee injuries in a left knee. (A) Anterior view with the left leg hanging off the table and at 90° flexion, anatomic landmarks drawn on skin before incision: contour of patella, contour of patellar tendon, tibial tuberosity, and planned skin incision anteromedial. (B) Anterolateral view with the leg hanging off the table and at 90° flexion, anatomic landmarks drawn on skin before incision lateral: Contour of fibular head, and planned skin incision posterolateral approach. (C) Anterolateral view with the leg hanging off the table and at 90° flexion, the common fibular nerve was dissected posterior to the biceps femoris tendon up to the fibular head, arrow for fibular (peroneal) nerve. (D) Anterolateral view with the leg hanging off the table and at 90° flexion, the graft was prepared central strip of Iliotibial band, arrow for iliotibial band graft.

Autografts from the bilateral ST and G tendons, and iliotibial band, are used. The knee is positioned in the supine position, and the ST and G tendons are harvested bilaterally through an anteromedial tibial approach (Figure 1A). The grafts are then measured for total length and thickness.

A posterolateral approach is performed on the knee ipsilateral to the injury, with the knee flexed at 90°. The incision is initiated at the lateral epicondyle and extended 10 cm proximally, 3 cm below the joint line, and 3 cm anterior to the fibular head (Figure 1B).

The common fibular nerve is dissected posterior to the biceps femoris tendon up to the fibular head. The fibular head is then dissected, identifying the FCL and the biceps femoris tendon (Figure 1C).

The LET uses a 12 mm‐wide and 15 cm‐long central strip of iliotibial band, the distal attachment to the Gerdy's tubercle is kept intact, while the iliotibial band is sectioned 10 cm proximal to the lateral epicondyle. The graft is prepared and sutured with nonabsorbable suture (Figure 1D).

Arthroscopy is performed through anterolateral and anteromedial portals of the knee, addressing meniscal and chondral injuries, as well as partial synovectomy for ACL reconstruction.

Reconstruction begins with the creation of the FCL tunnel on the lateral aspect of the fibular head, directed posteromedially and sized to match the ST and G tendons, ensuring protection of the common fibular nerve.

**FIGURE 2:**
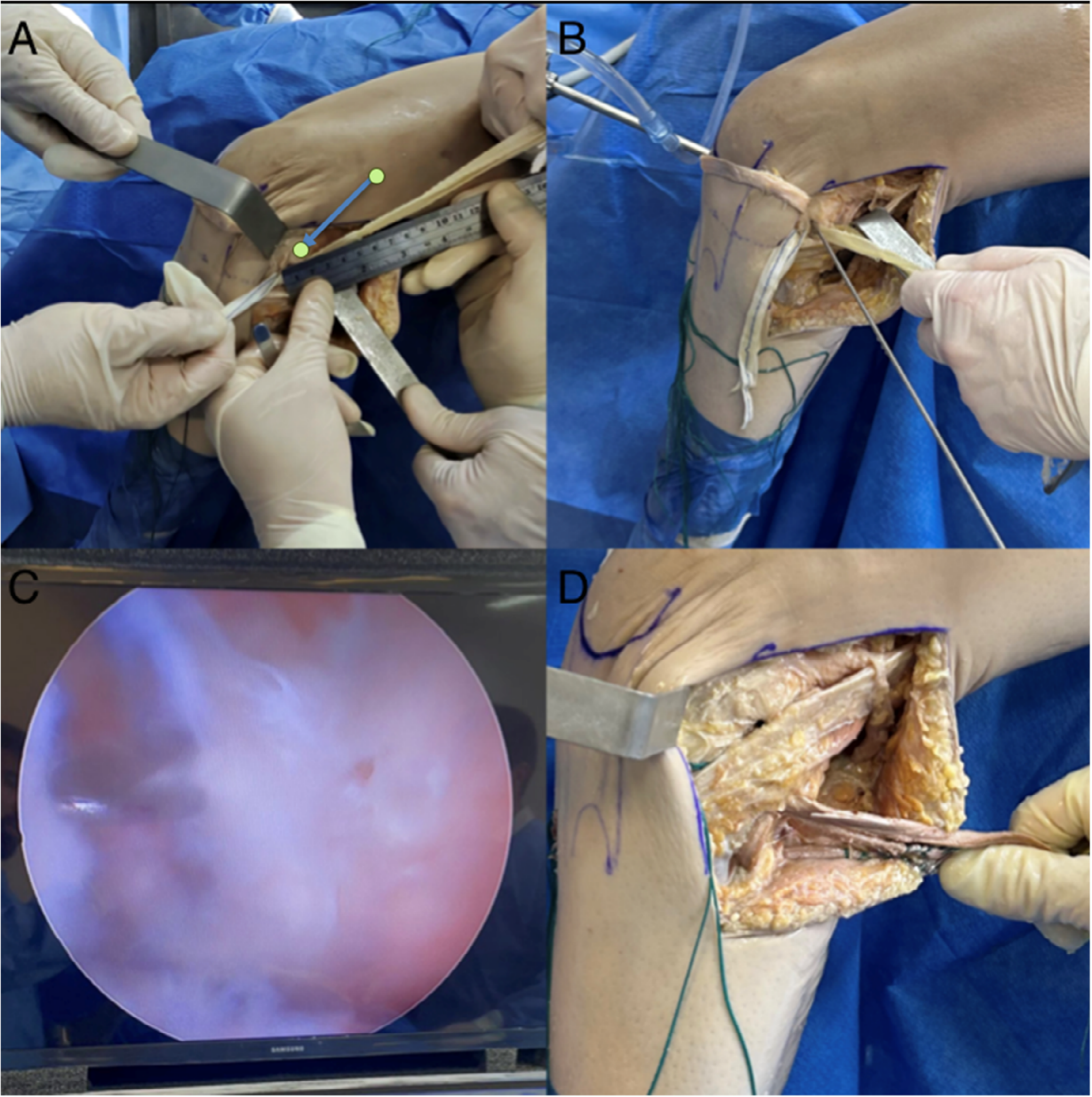
(A) Anterolateral view with the left leg hanging off the table and at 90° flexion, the lateral epicondyle was dissected to identify the insertion points of the fibular collateral ligament and the popliteus tendon blue arrow for lateral epicondyle. (B) Anterolateral view with the left leg hanging off the table and at 90° flexion, the isometric point of the lateral femoral condyle was identified. (C) Arthroscopic view through the anteromedial portal, the anatomic origin point of the anterior cruciate ligament in the femur was identified arthroscopically. (D) Anterolateral view with the left leg hanging off the table and at 90° flexion, the grafts of one ST and one G were passed through the fibular tunnel, passing deep to the iliotibial tract.

The lateral epicondyle is dissected to identify the insertion points of the FCL and the popliteus tendon. The latter is repaired with a nonabsorbable suture and dissected along its course up to the myotendinous junction, deep to the lateral head of the gastrocnemius (Figure 2A).

The popliteus tenodesis tunnel is drilled from anterior to posterolateral using a PCL tibial guide, starting anteromedial and distal to Gerdy's tubercle, exiting posteriorly 1 cm medial to the fibular head and 1 cm distal to the articular surface, in the region of the popliteus myotendinous junction. The tunnel diameter matches that required for 2 ST and 1 G tendon, with fibular nerve protection.

The isometric point of the lateral femoral condyle is identified as described by Stannard,^6^ located 6 mm anterior to the intersection of the FCL and popliteus tendon or at the intersection between the anterior line of the posterior femoral cortex and Blumensaat's line under fluoroscopy. This isometric test is conducted using a Kirschner wire at Stannard's point, with sutures placed in the fibular and popliteus tunnels to assess tension during 90° flexion and extension (Figure 2B).

Next, the anatomic origin point of the ACL in the femur is identified arthroscopically. The femoral tunnel is drilled from outside‐in using an angled ACL‐femoral guide at 95° approximately, introduced through the anterolateral portal and visualized via the anteromedial portal.^14^ The tunnel originated at the previously tested isometric point on the lateral wall of the lateral femoral condyle and extended to the anatomic point of the ACL on the medial wall of the lateral femoral condyle. Any outside‐in femoral guide can be used; a specific guide is not required for this technique as long as the isometric point on the lateral epicondyle and the anatomical point of the ACL is maintained, the angle can be adjusted as necessary for each piece of equipment. The tunnel diameter should be 1 mm larger than the combined diameter of 2 ST and 2 G tendons (Figure 2C).

The tibial ACL tunnel is created 7 mm anterior to the PCL insertion on the tibia, between the tibial spines and medial to the anterior horn of the lateral meniscus. It is important to test for impingement in full extension. The tunnel diameter should also be 1 mm larger than the combined diameter of 2 ST and 2 G tendons.

All five tendons (2 ST, 2 G, and the iliotibial tract) are passed from the femoral tunnel to the tibial tunnel using shuttle sutures. The ends of the ST and G grafts are positioned at the exit of the tibial tunnel. The iliotibial tract graft is pulled through the tibial tunnel, reinforcing the ACL reconstruction according to the Espejo‐Reina technique^15^ (Figure 2D).

**FIGURE 3:**
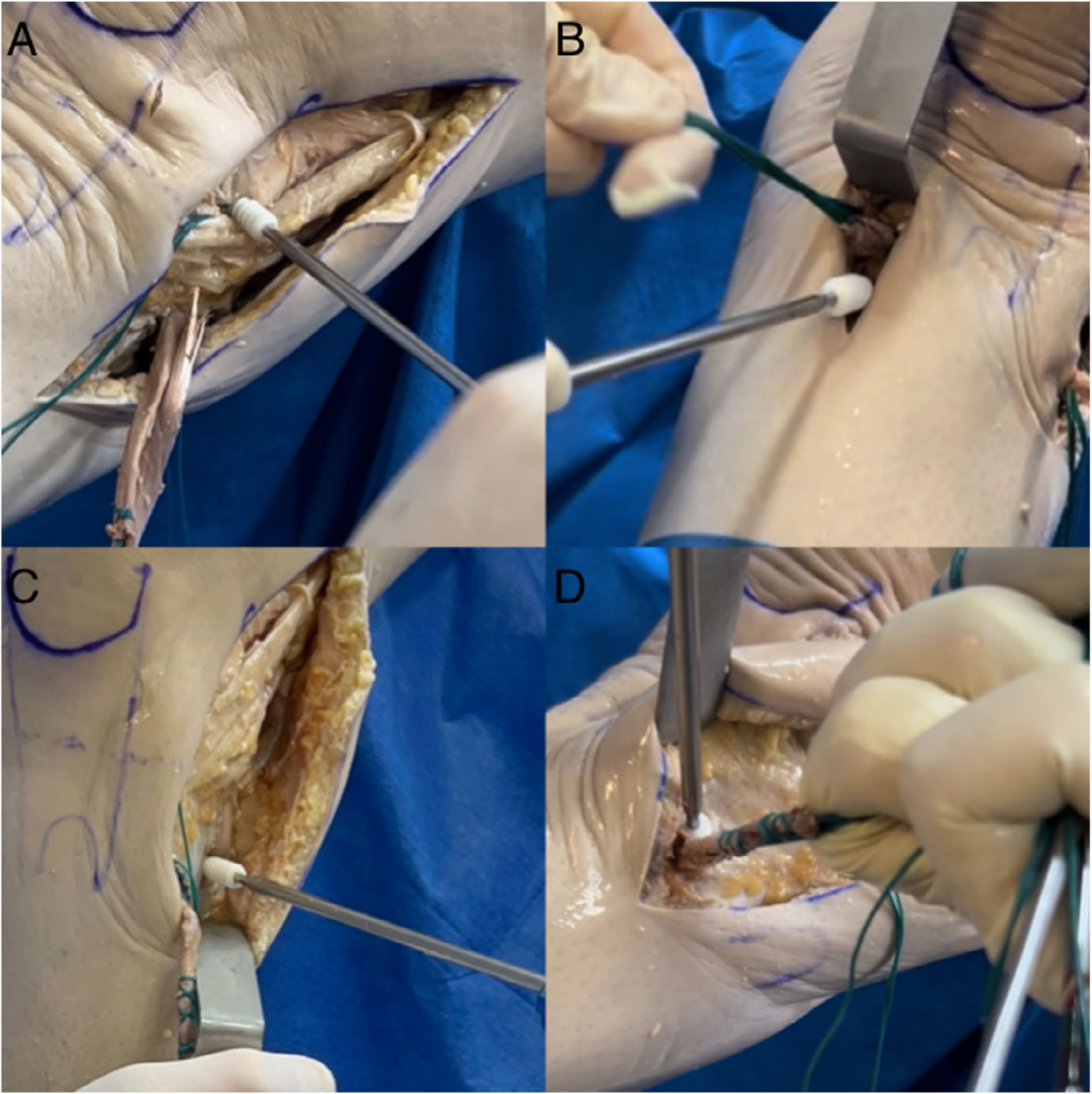
(A) Anterolateral view, fixation was initiated using a biocompatible interference screw in the femoral tunnel, with the knee at 30° of flexion and neutral rotation. (B) Anteromedial view, the 5 grafts were tensioned and fixed in the tibia with interference screw in full extension and neutral rotation. (C) Anterolateral view, the grafts of one semitendinosus and one gracilis tendons were passed through the fibular tunnel and fixed with the knee at 30° of flexion and in valgus. (D) Anterolateral view, the grafts of one semitendinosus and one gracilis tendons originating from femoral tunnel were passed through the posterolateral tibial tunnel, in conjunction with the semitendinosus graft originating from the fibula. Fixation was performed with the tibia in internal rotation, the knee at 30° of flexion and in valgus, using an interference screw inserted from anterior to posterior. Patient side: left leg.

Fixation is initiated using a biocompatible interference screw in the femoral tunnel, with the knee at 30° of flexion and neutral rotation—at this point, the anterolateral structure is being reconstructed, so the iliotibial tract should be tensioned (Figure 3A).

Next, the 5 grafts are tensioned and fixed in the tibia with another interference screw in full extension and neutral rotation (Figure 3B).

The grafts of 1 ST and 1 G are passed through the fibular tunnel from lateral to medial, passing deep to the iliotibial tract, and fixed with the knee at 30° of flexion and in valgus (Figure 3C).

The remaining ST + G graft is inserted into the popliteus tunnel, stabilizing the proximal tibiofibular joint, requiring a graft longer than 23 cm.

Finally, the grafts of 1 G and 1 ST originating from femoral tunnel are passed through the posterolateral tibial tunnel of the popliteus and tensioned from posterior to anterior, in conjunction with the ST graft originating from the fibula. Fixation is performed with the tibia in internal rotation, the knee at 30° of flexion and in valgus, using an interference screw inserted from anterior to posterior (Figure 3D).

The previously dissected popliteus tendon is then tensioned and integrated into the tenodesis at the lateral epicondyle. To ensure the sucess of this techinique, several pearls and pitfalls are described in Table 2.

**TABLE 2:** Pearls and Pitfalls

Pearls
Perform meticulous dissection and protection of the common peroneal nerve during the creation of bone tunnels.	
Suture and clearly differentiate the iliotibial band, as it will require traction during femoral screw insertion.	
Assess the isometry of the femoral tunnel to ensure optimal graft positioning and function.	
Perform popliteus tendon tenorrhaphy at the location of the femoral tunnel to improve posterolateral corner stability.

Pitfalls	
The common peroneal nerve may present with altered anatomical positioning in multiligament knee injuries; initiate dissection from proximal to distal to minimize the risk of iatrogenic injury.	
Evaluate the possibility of fibular head fracture.	
Exercise caution during the creation of the tibial tunnel for the popliteus tendon; the tunnel should be initiated medial to Gerdy's tubercle to avoid structural compromise.	
Delayed initiation of rehabilitation may hinder the recovery of range of motion, adversely affecting functional outcomes.	

## DISCUSSION

The use of a single isometric femoral tunnel, rather than three separate tunnels for the ACL, popliteus tendon, and FCL, reduces complications such as tunnel collision, trochlear injury, and osteonecrosis. It also shortens surgical time, lowers costs, and eliminates the need for long grafts, an especially valuable feature when allografts are not available.

The technique described is more reproducible and safer than creating two parallel tunnels spaced 18.5 mm apart as suggested by LaPrade,^8^ along with a third ACL tunnel, avoiding collisions, especially considering that this distance varies with patient size. The Korean study by Kim et al.^12^ reported that the distance between the femoral insertions of the FCL and popliteus tendon is approximately 9.3 mm, significantly smaller than LaPrade^8^ described, making the creation of two separate femoral tunnels unfeasible.

Conventional techniques aim to replicate the function of the popliteus muscle through static tenodesis.^2^, ^6^, ^8^, ^9^ However, the popliteus is a dynamic stabilizer of internal rotation and posterior tibial translation and also participates in the movement of the lateral meniscus. Therefore, reintegrating the popliteus tendon into the reconstruction is essential for proper knee function.

Including anterolateral tenodesis in the combined reconstruction of the ACL and PLC using a single femoral tunnel and autografts increases rotational stability and resistance to varus stress. It also allows the use of a thicker and more effective iliotibial band graft to reinforce the ACL.

This technique provides exceptional lateral compartment stability through the so‐called “lateral tripod”: the posterolateral base with popliteus tenodesis, the lateral pillar with FCL reconstruction, and the anterolateral column with the iliotibial tract, having the ACL as the central pivot.

## DISCLOSURES

The authors (I.N.P., R.E.P., I.S.M.P., J.G.C.C.V., N.S.L.R., R.M.C.P., V.C.O., G.M.P.B., K.E.L.C., R.F., A.M.V.) declare that they have no known competing financial interests or personal relationships that could have appeared to influence the work reported in this article.
